# Exosomes secreted by *Blastocystis* subtypes affect the expression of proinflammatory and anti-inflammatory cytokines (TNFα, IL-6, IL-10, IL-4)

**DOI:** 10.3389/fmed.2022.940332

**Published:** 2022-08-11

**Authors:** Mojtaba Norouzi, Majid Pirestani, Ehsan Arefian, Abdolhossein Dalimi, Javid Sadraei, Hamed Mirjalali

**Affiliations:** ^1^Department of Parasitology, Faculty of Medical Sciences, Tarbiat Modares University, Tehran, Iran; ^2^Department of Microbiology, School of Biology, College of Science, University of Tehran, Tehran, Iran; ^3^Foodborne and Waterborne Diseases Research Center, Research Institute for Gastroenterology and Liver Diseases, Shahid Beheshti University of Medical Sciences, Tehran, Iran

**Keywords:** exosomes, *Blastocystis* subtypes, proinflammatory, anti-inflammatory, cytokines

## Abstract

**Background:**

*Blastocystis* sp. is a common intestinal parasite, possibly responsible for diarrhea, vomiting and nausea, abdominal pain, and irritable bowel syndrome. However, many studies focused on this issue due to the uncertainty of its pathogenic potential. The extracellular vesicles (EVs) are significant mediators for cellular communication, carrying biological molecules such as proteins, lipids, and nucleic acids. Compared with other parasites, little is known about the *Blastocystis* EVs. Hence the present investigation was done.

**Methods:**

The *Blastocystis* parasites were cultured in the DMEM medium, and a 550–585 bp fragment was amplified using PCR, and sequencing was done. A commercial kit was used for exosome extraction and dynamic light scattering (DLS), flow cytometry (CD63, CD81 markers), and electron microscopy tests to determine their morphology. The human leukemia monocytic cell line (THP-1) was exposed to *Blastocystis* EVs. Next, the expression of proinflammatory and anti-inflammatory cytokines, including IL-4, IL-6, IL-10, and tumor necrosis factor-alpha (TNF-α), were measured using quantitative PCR.

**Results:**

Exosomes were extracted from ST1-3 *Blastocystis* sp. According to the DLS assay, the size of the exosomes was in the range of 30–100 nm. Electron microscopy images and CD63 and CD81 markers also confirmed the exosome's size, structure, and morphology. According to real-time PCR results, ST1-derived exosomes caused IL-6 and TNF-α upregulation and IL-10 and IL-4 downregulation, ST2- and ST3-derived exosomes downregulated IL-10, and ST3-derived exosomes caused IL-6 upregulation. There is a statistically significant difference (*P* ≤ 0.05).

**Conclusion:**

To our knowledge, this is the first report of the release of exosome-like vesicles by the human parasite, *Blastocystis*, and the provided information demonstrates the role of this parasite, particularly ST1 on proinflammatory and anti-inflammatory cytokines and navigating the host response.

## Introduction

Parasitic diseases contribute to high morbidity and mortality yearly, particularly in developing countries (1). The parasitic agents employ many molecular mechanisms to communicate and manipulate the host responses (2). The extracellular vesicles (EVs) have been recognized as novel mediators in maintaining intestinal homeostasis, principally in the intestinal mucosa (3). These nano-molecules are also implicated in coordinating the growth and development, horizontal gene transfer, and host-pathogen communications, particularly at the early stages of the infection. Several mechanisms exist for EV uptake by host cells (4, 5). Phagocytosis and macropinocytosis are primarily involved in EV uptake, surrounded by the plasma membrane (6). Other entry routes are receptor-mediated contact, fusion with the target cell plasma membrane, and delivery of the soluble cargo to the cells. Reportedly, the information carried by EVs isis involved in disease development (7). EVs have been known to possess clinical applications, both as diagnostic biomarkers and therapeutic agents (8).

There are different types of EVs based on size and biogenesis. Exosomes (30–100 nm) are composed of late endosomes containing vesicles that combine with the plasma membrane, (9) and are released into the extracellular environment, microvesicles (100–1,000 nm) are directly germinated from the plasma membrane and are produced by outward budding of the plasma membrane and apoptotic bodies (1,000–5,000 nm) result from the interaction of myosin-actin during programmed cell death or apoptosis and contain cell contents such as organelles (10). Specific proteins named calpins with cysteine-dependent calcium protease activity, found in most eukaryotes and some prokaryotes, are involved in the releasing processes of EVs (11). In parasitic agents, the EVs may play a critical role in modulating the host's immune responses (12, 13). The following proteins are consistently found within exosomes: heat shock proteins (HSP60, HSP70, and HSP90), nucleic acids (DNA, RNA, microRNA), tetraspanins (CD81, CD63, and CD37), anxins (I, II, V and VI), cytoskeletal proteins (actin and tubulin), metabolic enzymes, proteins involved in translation (elongation factors 1 and 2) and signaling proteins (14, 15). These biological macromolecules are particularly interested in the diagnosis of infectious disease pathology (16).

The extracellular protozoan parasite, *Blastocystis* sp., is a gastrointestinal inhabitant in animals and humans, being assigned to cause abdominal pain, diarrhea, vomiting, and nausea. It has also been speculated to be involved in the initiation and or progression of irritable bowel syndrome (IBS) (17, 18), inflammatory bowel disease (IBD), and colorectal cancer (19). The phylogenetic analysis has placed the *Blastocystis* within the Stramenopiles group, along with brown algae (20). Among 28 identified subtypes (STs) of *Blastocystis*, nine (ST1-ST9) have been found in humans (21). Based on the published literature, subtypes 1–3 have been linked to chronic urticaria and skin disorders (1, 22, 23). Moreover, *Blastocystis* can activate cytokine production and immune responses *in vitro* (24). Cysteine protease enzymes in *Blastocystis* (especially ST7) can induce myosin light chain phosphorylation, ZO1 protein degradation, and F-actin reorganization in the Caco-2 cell line (25, 26). Compared with other parasites, little is known about the characterization, function, and host-parasite interaction of the *Blastocystis* EVs (27). Therefore, the present experiment investigated the effects of the *Blastocystis* exosomes on the expression of proinflammatory and anti-inflammatory cytokines such as IL-6, TNFα, IL-4, and IL-10 using molecular techniques.

## Materials and methods

### Parasite culture

**Figure 1:**
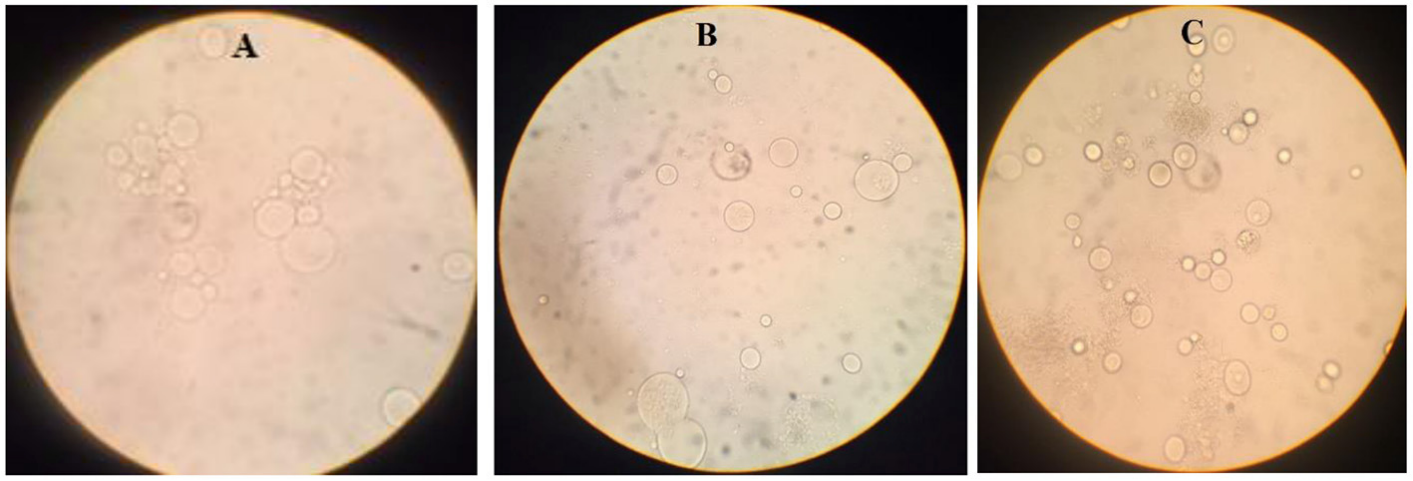
*Blastocystis* sp. in the medium (DMEM) with FBS and penicillin-streptomycin [**(A)**: Subtype 3, **(B)**: Subtype 2, **(C)**: Subtype 1].

Positive *Blastocystis* samples were cultured in Dulbecco's Modified Eagle medium (DMEM) supplemented with 10% fetal bovine serum (FBS) and Penicillin-Streptomycin (1,000-unit penicillin and 4 mg/ml streptomycin) (Figure 1).

### DNA extraction, polymerase chain reaction, and sequencing

**Figure 2:**
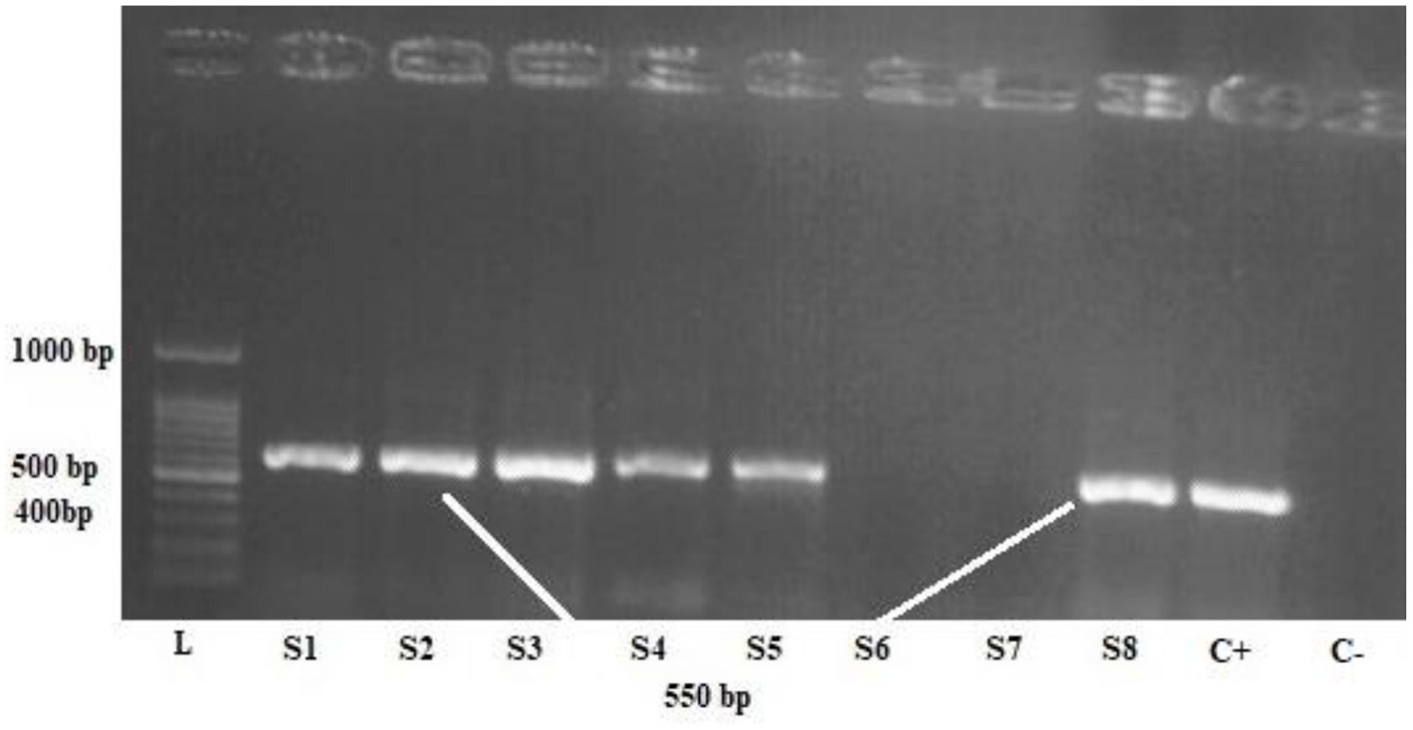
One percent agarose gel electrophoresis of the polymerase chain reaction product of *Blastocystis* samples, L: 100-bpDNA ladder, C + positive control, C- negative control, S1,2,3,4,5,8 positive samples.

Upon propagation of the parasites in the culture medium, DNA was extracted using DNA Extraction Kit (SINACLON), based on the manufacturer's protocol, and a ~600 bp fragment of the *Blastocystis* small subunit ribosomal DNA (SSU rDNA) gene was amplified using PCR method, according to the previous studies (Figure 2) (28). The amplification was done using a forward primer, RD5 (ATCTGGTTGATCCTGCCAGT) (29), and a reverse primer, BhRDr (GAGCTTTTTAACTGCAACAACG) (30).

The PCR amplicons were run on a 1% gel electrophoresis and visualized using ultraviolet (UV) illumination in a Gel-Doc apparatus. Sequencing was performed by the Sanger method. All obtained sequences were edited by SEQUNCHERTM software (ver. 5.4.5), and a comparison with other sequences deposited in the GenBank was performed using the basic local alignment tool (BLAST), available at http://blast.ncbi.nlm.nih.gov. Next, subtypes 1, 2, and 3 of *Blastocystis* sp. were selected for exosome extraction and further evaluation.

### Exosome extraction

Initially, an FBS-free DMEM medium was used to culture the parasites for 48 h before extraction. A commercial kit, Exocib (Cib Biotech Co., IRI), was used for exosome extraction, and the extraction steps were done based on the manufacturer's guidelines. The purified exosomes were stored at 4°C for a few days or kept at −20°C or −80°C for a long time.

### Quantification of exosome concentration

For this purpose, a kit-based Bradford test (Diba NoAvaran Azma Company, DNAbiotech) was used. The standard bovine serum albumin (BSA) was diluted with 1 mg/ml storage solution (100 μl BSA + 900 μl PBS or distilled water) and prepared based on the dilution kit protocol. A standard graph was plotted, and the trendline, the line equation, and the regression coefficient were determined on the graph. Finally, 20 μl of each sample was mixed with 180 μl of the reagent, and the optical density (absorbance) was read at 595 nm after 10 min.

### Exosome characterization

#### Dynamic light scattering

This procedure was done to determine the extracted exosomes' size distribution. For this aim, 50 μl of exosome sample for each subtype of interest was diluted in 950 μl of PBS. Then, the size distribution of the exosomes was read by Zetasizer Nano (ZS (Malvern Instruments, Southborough, UK) at 25 ° C with a refractive index of 1.38 and absorption of 0.01.

#### Evaluation of exosome surface markers by flow cytometry technique

The exosome surface markers, CD63 and CD81, were evaluated using the flow cytometry technique. Initially, the exosomes were connected to sulfate aldehyde sulfate beads (size: 4 μm) to improve their size for the reader device. Briefly, 30 μg of the exosomes from each subtype was incubated with 90 μl of bead for 95 min at ambient temperature. Subsequently, the volume of the samples was increased to 1 ml with PBS and placed on a test tube rotator at 4°C for 16 h. Then the attachment process was terminated by adding 110 μl of 100 mM glycine solution and incubating for 30 min at room temperature. The exosome-coated beads were triple washed with PBS containing 0.5% BSA. Then, the exosome-bead complex was incubated with anti-CD63 and anti-CD81 antibodies separately for each group for about 1 h at 4°C. In the final step, the expression of the mentioned markers was examined by the flow cytometry method.

### Evaluation the size, structure, and morphology of exosome

#### Scanning electron microscopy

The morphology and size of the exosomes were also monitored using SEM imaging. First, the samples were fixed using pre-cooled 2.5 % glutaraldehyde solution (Sigma–Aldrich, Germany), placed on the specimen stub, and snap-frozen in a freeze-dryer (Model 5006; Dena Vacuum Industry Co., Ltd). Then, the specimens were sputter-coated with gold-palladium and imaged by the SEM system.

#### Transmission electron microscopy

Purified exosomes were diluted with an equal volume of 4% paraformaldehyde at 4°C for 30 min, then carefully placed on a carbon-coated 300-mesh copper grid for 20 min, followed by a fixation step using 1% glutaraldehyde for 5 min. The mesh was contrasted with 2% uranyl acetate, washed twice, and the morphology of the isolated exosomes was observed. Exosome images retrieved through TEM (TEM, LEO 906, Zeiss, Germany) were analyzed by ImageJ software.

### Expression of inflammatory and proinflammatory cytokines in the presence of exosomes

#### Cell culture

**Figure 3:**
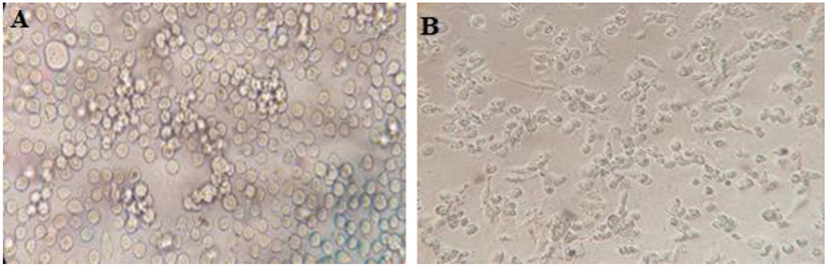
**(A)** THP-1 cell in RPMI 1,640 medium and **(B)** THP-1 macrophages in RPMI 1,640 medium.

The Roswell Park Memorial Institute (RPMI 1,640) medium supplemented with 10% FBS, 1% penicillin/streptomycin, and 1% L-glutamine was used to culture the human leukemia monocytic cell line (THP-1) (Figure 3). The cells were enumerated with trypan blue (0.4%), and 400 × 10^3^ cells were seeded in each well of 12-well plates and subsequently treated by 50 ng phorbol myristate acetate (PMA) overnight, then washed three times. The cells were then rested for a day.

##### Exposure of THP-1 cells to exosomes

Prepared THP-1 cells were exposed to 10 μg of exosomes from each subtype, being incubated for 24 h. Next, the cells were trypsinized and collected in a microtube for the next steps.

#### RNA extraction

RNA extraction was performed manually according to the following protocol: First, 1 ml of Trizol (Thermo Scientific) was added to the collected samples and a pipette, then 250 μl of chloroform was added and mixed. The next step was incubated at room temperature for 15 min, then centrifuged at 15,000 rpm at 4° C for 15 min. In the next step, the supernatant was removed and transferred to a new microtube, then 600–800 μl of cold isopropanol was added to it and mixed together. The samples were kept at −20 °C for 24 h. They were then centrifuged at 13,000 rpm at 4 °C for 45 min. The supernatant was then removed, and 1 ml of 70% cold ethanol was added to the RNA precipitate (washing). The samples were centrifuged at 20,000 rpm at 4°C for 20 min. Remove the supernatant and allow the precipitate to dry for about 10 min (drying). Then 20 μl of sterile distilled water was added to the precipitate and gently pipetted to dissolve the RNA precipitate in water. The extracted RNA was stored at −70°C. The quality and concentration of RNA were evaluated with a nanodrop spectrophotometers (ND2000, Thermo Scientific). The OD 260/280 nm ratio was reported to be about 2.00.

### CDNA synthesis and real-time PCR

For cDNA synthesis, total RNA was reverse-transcribed using M-MLV reverse transcriptase according to the manufacturer's instructions (RT-ROSET Kit). Real-time PCR was performed in triplicate using 50 ng of cDNA with RealQ Plus 2x Master Mix Green reagent (Ampliqon) in a Rotor Gene-Q thermal cycler (Qiagen). Primer sequences are shown in Table 1. Relative gene expression values were normalized to β-actin and calculated using the comparative CT method (2^∧^–ΔΔCT). The values are presented as mean n-fold differences compared to the control (*P* ≤ 0.05 was reported to be significant).

**Table 1:** List of primers were used in real-time PCR.

**No**.	**Gene**	**Sequence**		**Ref**
1	IL4	5′-ACAGCCTCACAGAGCAGAAGACT-3′	Forward	(31)
		5′-TGTGTTCTTGGAGGCAGCAA-3′	Reverse	(31)
2	IL-10	5′-GCAGTGGAGCAGGTGAAGAG-3′	Forward	(32)
		5′-CGGAGAGAGGTACAAACGAGG-3′	Reverse	(32)
3	IL-6	5′-AGTTGCCTTCTTGGGACTGA-3′	Forward	(31)
		5′-TCCACGATTTCCCAGAGAAC-3′	Reverse	(31)
4	TNF-α	5′-GAACTGGCAGAAGAGGCACT-3′	Forward	(32)
		5′-AGGGTCTGGGCCATAGAACT-3′	Reverse	(32)
5	β-actin	5′-GCCATGTACGTAGCCATCCA-3′	Forward	(33)
		5′-ACGCACGATTTCCCTCTCAG-3′	Reverse	(33)

## Result

### Subtyping of the *Blastocystis sp*.

**Figure 4:**
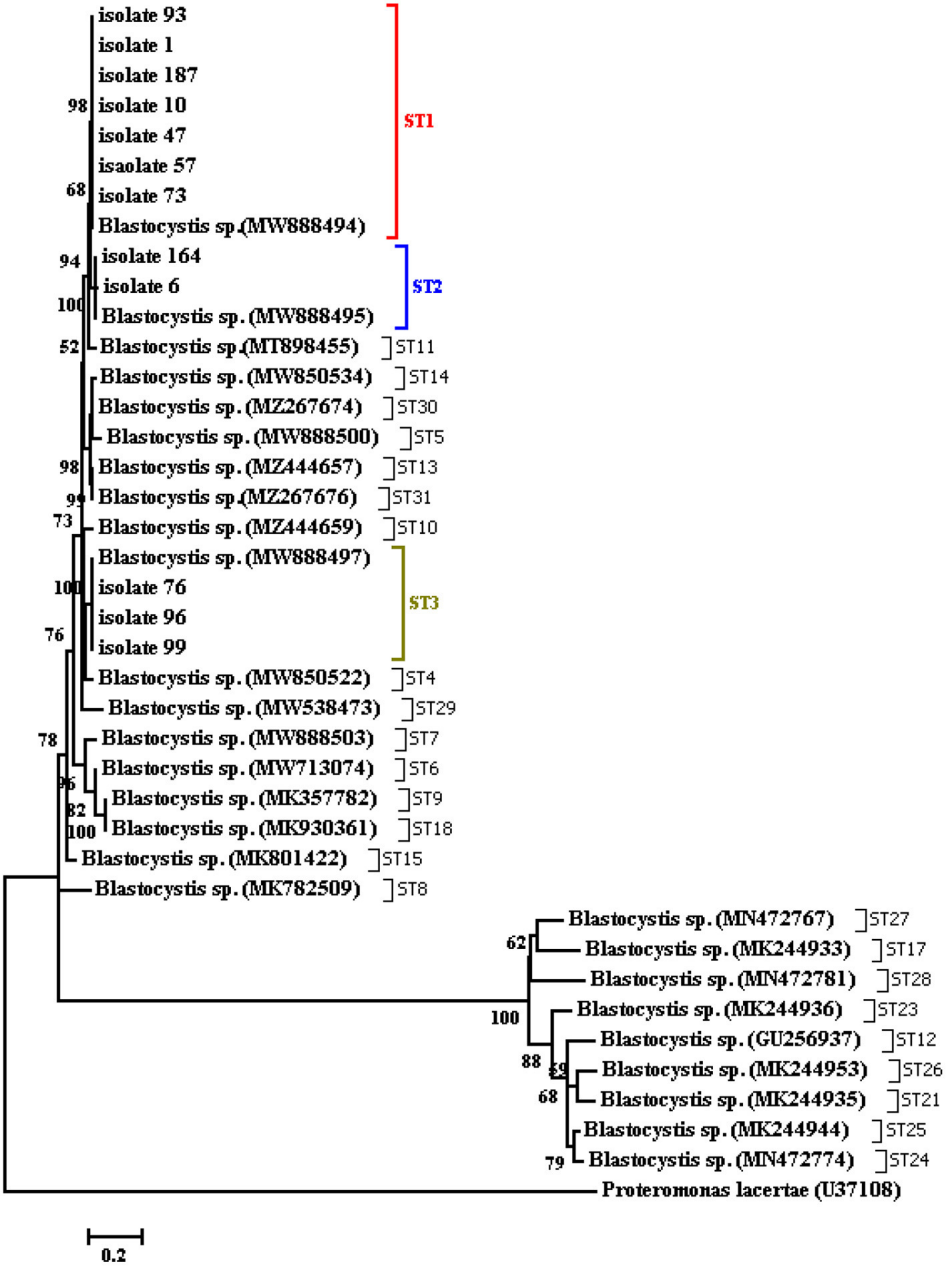
Phylogenetic tree of the SSU-rDNA gene sequences of *Blastocystis* isolates constructed by the neighbor-joining method using Mega4 software.

The *Blastocystis* parasites were cultured in a DMEM medium enriched with 10% FBS and penicillin-streptomycin antibiotics, and a molecular investigation followed by sequencing of the propagated parasites revealed ST1-ST3. The sequences have been deposited in GenBank under accession numbers OL457221-OL457257. Multiple sequence alignments were performed using the ClustalW, and phylogenetic analyses using the maximum-likelihood (ML) method were carried out using the MEGA7 using all subtypes of *Blastocystis*. The tree was constructed (500 replicates) using the 18S rRNA gene sequence of Proteromonas laceratae (U37108) as an outgroup (Figure 4).

### Determination of the concentration of extracted exosomes

Based on the Bradford assay, the concentrations of the exosomes for subtypes 1, 2, and 3 included 1.9 μg/μl (ST1), 2.3 μg/μl (ST2), and 2.2 μg/μl (ST3).

### Confirmatory tests for exosome extraction

**Figure 5:**
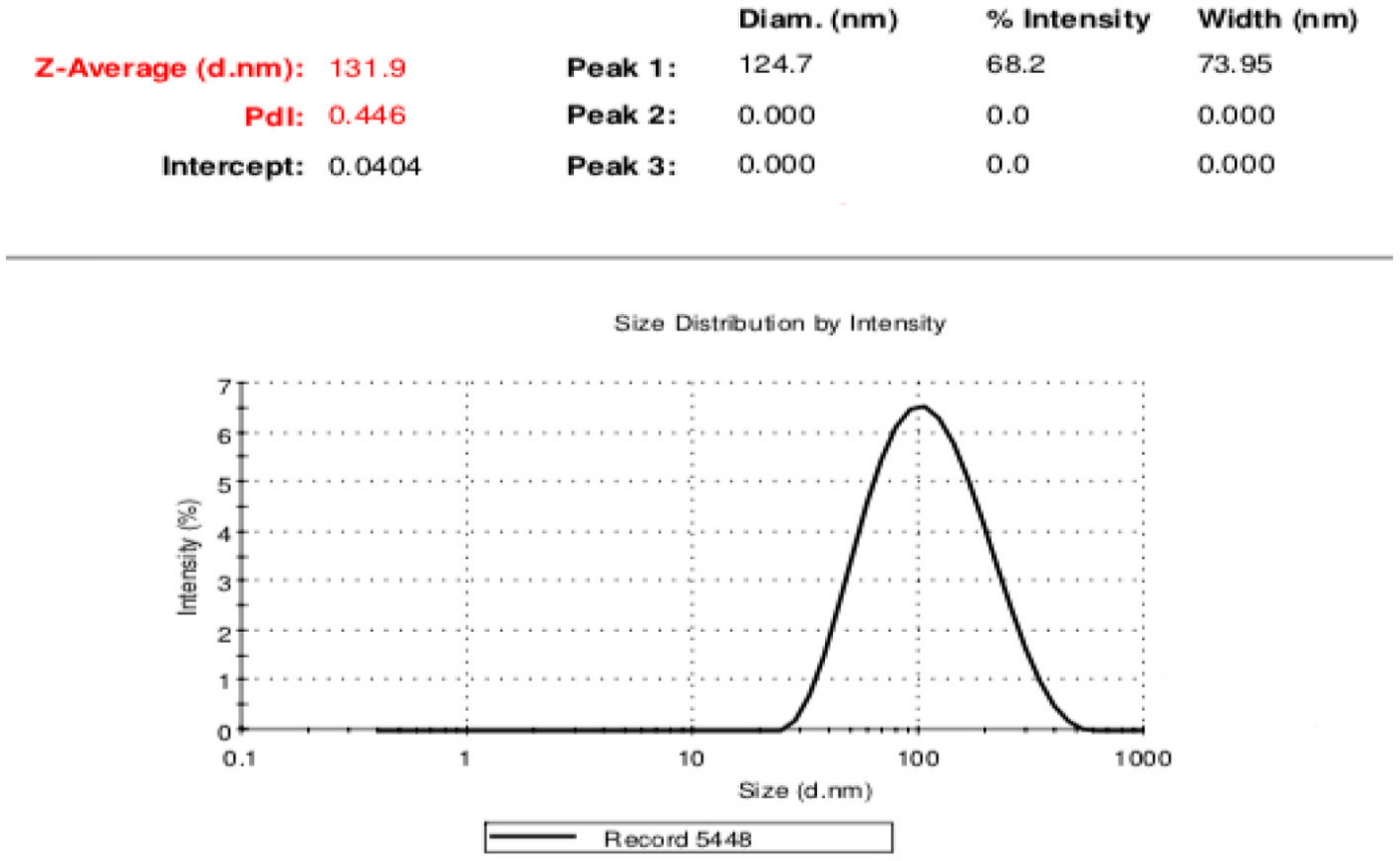
Extracellular vesicle size distribution using DLS techniq.

**Figure 6:**
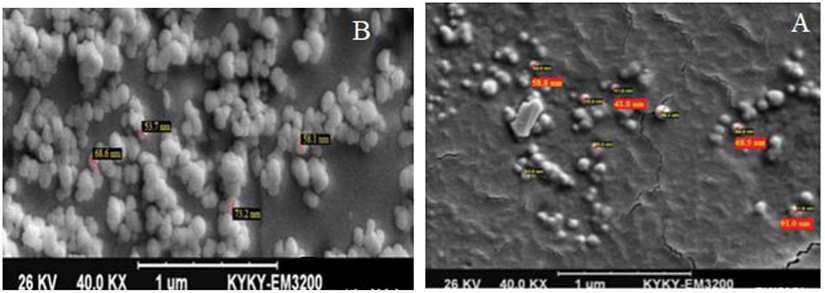
SEM electron microscope image of exosome specimens of *Blastocystis* subtypes [**(A)**: Exosomes without conjugation and **(B)**: with sulfate aldehyde sulfate beads].

**Figure 7:**
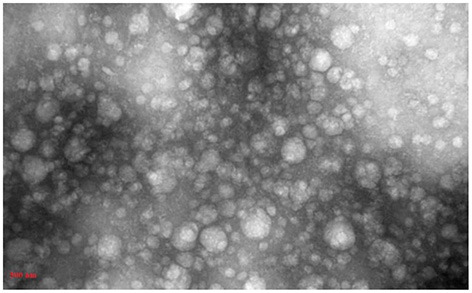
TEM electron microscope image of exosome specimens of *Blastocystis* subtypes.

**Figure 8:**
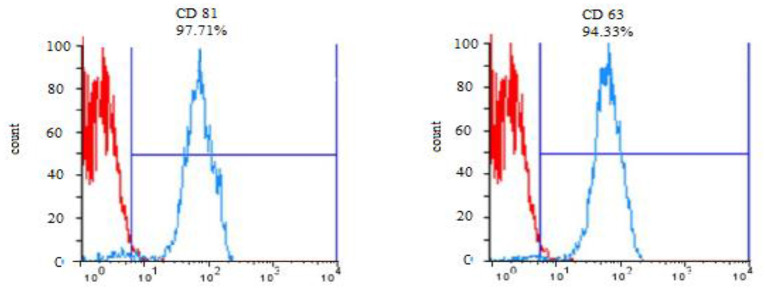
Expression of CD 63 and CD 81 surface markers in exosomes of *Blastocystis* subtypes.

DLS test was performed to determine the size of the exosomes. The peak of EVs population was obtained in the range of exosomes, and the intensity was 68.2% (Figure 5). Furthermore, the electron microscopy studies confirmed the homogeneity of the population with a size of 30–100 nm and their spherical morphology (Figures 6, 7). Also, due to existing limitations, we were not able to measure exosomes in the range of 100–1,000 nm. The flow cytometry results showed the expression of CD63 (94.33%) and CD81 (97.71%) markers on the surface of exosomes (Figure 8).

### Expression of inflammatory and proinflammatory cytokines by THP-1 cells exposed to the exosomes

**Figure 9:**
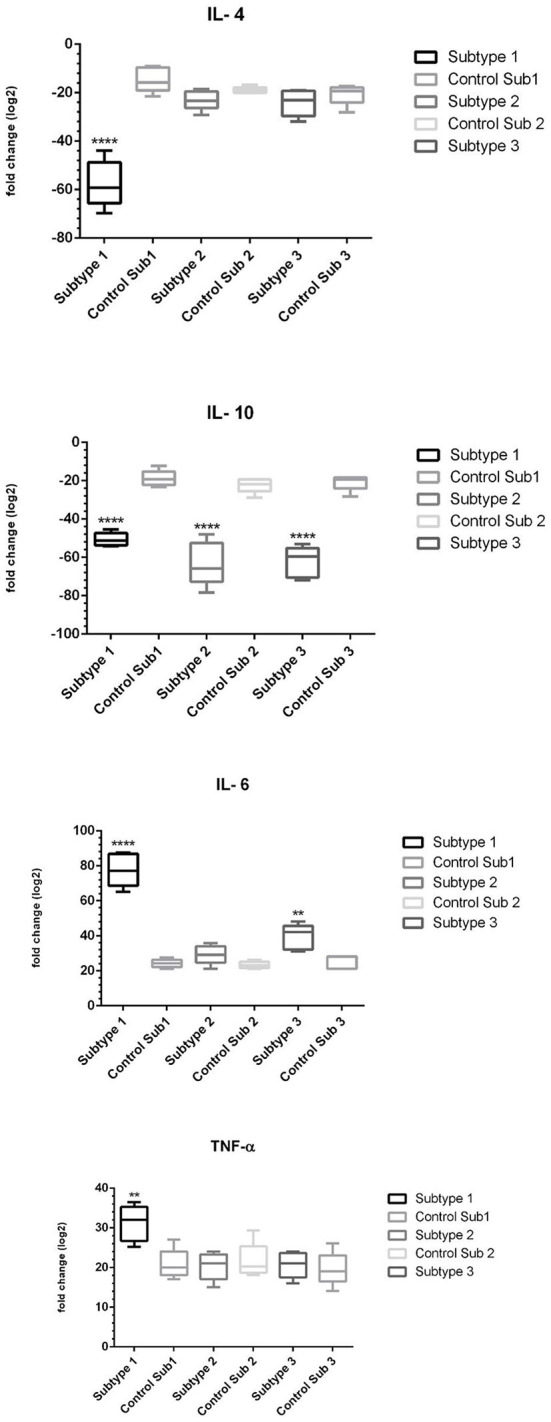
The expression of proinflammatory and anti-inflammatory cytokines in the presence of exosomes of subtypes 1, 2 and 3 of *Blastocystis* (The sign (*) in each subtype indicates which cytokine was statistically significant compared to control) (**p < 0.01; ****p < 0.0001).

Next, the exosomes were exposed to THP-1 cells for 24 h, then cells were trypsinized, and RNA extraction, cDNA synthesis, and real-time PCR experiments were performed. Analysis of real-time PCR output and fold change calculation showed (Table 2) that in ST1, the expression of IL-6 and TNF-α was upregulated, compared to the control group. Also, ST1 showed a decrease in IL-4 and IL-10 expression, while IL-4 expression was not changed in ST2- and ST3-exposed THP-1 cells (Figure 9). It is noteworthy that IL-10 showed a decrease in expression in all three subtypes compared to the control group Notably, The expression of TNF-α in exosome-exposed cells in subtype 1 was upregulated compared to the control (LPS).

**Table 2:** Results of real-time PCR data analysis.

**Exosome**	**Cytokine**	**Expression**	**Fold change**	***P*-value**
**Subtype 1**	IL-4	Down Regulate	−67.323	<0.0001
	IL-10	Down Regulate	−53.259	<0.0001
	IL-6	UP Regulate	87.620	<0.0001
	TNFα	Not different	36.422	0.004
**Subtype 2**	IL-4	Not different	−19.849	0.999
	IL-10	Down Regulate	−78.385	<0.0001
	IL-6	Not different	35.677	0.580
	TNFα	Not different	22.576	0.994
**Subtype 3**	IL-4	Not different	−19.578	0.908
	IL-10	Down Regulate	−68.967	<0.0001
	IL-6	Down Regulate	43.188	0.002
	TNFα	Not different	23.287	0.998

*The values are presented as mean n-fold differences compared to the control (P-value ≤ 0.05 was reported to be significant)*.

## Discussion

*Blastocystis* is an extracellular organism in the gut of animals and humans, which can suppress the inducible nitric oxide (iNOS) production and cleave the immunoglobulins, evading the host's immune system responses (34). This compromised molecular milieu also paves the way for other co-infections to infect the intestinal epithelium and cause disease (35). During the last decades, the molecular basis of the host-parasite cross-talk has been more elucidated, highlighting to be mediated by the EV nano-molecules (~30–5,000 nm) as tiny vehicles for cellular communication that may carry proteins, lipids, nucleic acids, and metabolites from their cellular origin, essential for genetic exchange, biomarker identification, and the diagnosis of diseases (36, 37).

Based on the *in vitro* experiments, *Blastocystis* parasites can elicit cytokine production and immune responses (38). Despite the availability of some *Blastocystis* subtype's genomes, our knowledge is still in its infancy on the biological and pathological mechanisms of the parasite (39). Curiously, membrane proteins and the integrity of the epithelial barrier could be substantially disrupted by the *Blastocystis* ST7 *via* degradation of tight proteins (3). So far, few studies have characterized EVs in *Blastocystis*, and the early evidence was provided by Tan through the TEM experiment (35). According to recent studies, *Blastocystis*-derived EVs were identified in ST7 B and H isolates, with cup-shaped morphology and an average size of 50 to 240 nm consistent with EVs derived from other parasites (40).

In recent years, a great deal of interest has been focused on the epidemiology, phylogeny, and cell biology of *Blastocystis*; only a few studies have addressed the parasite virulence and specific host responses (41). Based on the published literature, gastrointestinal symptoms similar to IBS, including diarrhea, abdominal pain, constipation, nausea, inflammation, and edema, have been reported in patients infected with *Blastocystis* (42). On the other hand, the parasite has been recognized mainly in asymptomatic individuals, thus possibly being non-invasive (43). Edema and infiltration of the inflammatory cells into the lamina propria have been reported in the cecum and colon of infected mice (44). An experiment showed that *Blastocystis* induces IL-8 production in colon T84 epithelial cells in a time-dependent manner, which provokes the inflammatory cells to invade the intestinal mucosa, resulting in tissue damage and gastrointestinal disorders (45).

Previously, no investigation was done to evaluate the association between IL-6 and TNF-α gene polymorphisms in susceptibility to diseases such as IBS (46). Here, we evaluated such an association and revealed a significant association between IL-6 gene expression in THP-1 cells exposed to ST1 exosomes. This may suggest that the presence of a parasite such as *Blastocystis* would play a substantial role in IBS by promoting the clinical outcomes. Another major finding in the current study was that no significant difference was observed regarding TNF-α expression in comparison with the control group, which is inconsistent with Iguchi et al. study, demonstrating significant upregulation of IFN-γ, IL-12, and TNF-α cytokines in the cecal mucosa (47). Downregulation of IFN-γ and TNF-α, along with the upregulation of IL-6 and IL-8, has been observed in colorectal cancer (48). This evidence indicates that the *Blastocystis* antigen (Blasto-Ag), an example of the extracellular allergen, has stimulated the humoral responses, leading to inflammatory reactions and cell propagation to combat the infection (49). Another study by Yakoob et al. demonstrated significantly lower IL-10 levels and colonic eosinophilic infiltration associated with IL-8 in the *Blastocystis* ST1 compared to ST3 and control (32). In the current study, we found a reduction in the expression of IL-10 in ST1, ST2 and ST3. It has been recognized that IL-4 and IL-13 cytokines mediate the goblet cell hyperplasia during the gut infection, which the latter plays a significant protective role against the infection (50). Our results demonstrated a decrease in IL-4 and IL-10 expression and an increase in IL-6 and TNF-α expression in *Blastocystis* ST1. Reportedly, IL-10 is essential in regulating inflammatory responses, as it reduces the production of chemotactic factors such as IL-8 (51). Altogether, the increased mucin layer and fluid secretion, goblet cell hyperplasia, and enhanced intestinal propulsive activity result in the expulsion of noxious agents from the gut lumen (52).

Finally, the results presented here highlighted the significance of the *Blastocystis* ST1 exosomes on the expression of IL-6, IL-10, IL-4 and TNF-α cytokines, strengthening the hypothesis that the *Blastocystis* parasite is a potent contributor to the inflammatory processes. To better understand the interaction of EVs of *Blastocystis*, other inflammatory cytokines such as MCP-1 and IL1B should be investigated. However, the actual pathogenicity of *Blastocystis* and its association with gastrointestinal symptoms is yet to be determined through extensive research, possibly in the field of EVs, their biogenesis, uptake, and cellular communication.

## Conclusion

To our knowledge, this is the first report of the release of EVs by the human parasite *Blastocystis*, and our data demonstrate the role of this parasite, particularly ST1, on proinflammatory and anti-inflammatory cytokines and navigating the host responses. Further studies on the arrangement and function of these biologically-active vesicles could assist us in developing unprecedented therapeutic strategies, opening new doors toward the role of the *Blastocystis* parasite in gastrointestinal diseases.

## Data availability statement

The original contributions presented in the study are included in the article/supplementary material, further inquiries can be directed to the corresponding author.

## Ethics statement

The studies involving human participants were reviewed and approved by Tarbiat Modares University. The patients/participants provided their written informed consent to participate in this study.

## Author contributions

MP and MN designed the study. EA and MP contributed to the methodology. EA and HM analyzed the data. AD, JS, and MN reviewed and edited the manuscript. EA was responsible for advising on controversial issues. MP is responsible for the overall content as a corresponding author. All authors have read and approved the final version of the manuscript.

## Conflict of interest

The authors declare that the research was conducted in the absence of any commercial or financial relationships that could be construed as a potential conflict of interest.

## Publisher's note

All claims expressed in this article are solely those of the authors and do not necessarily represent those of their affiliated organizations, or those of the publisher, the editors and the reviewers. Any product that may be evaluated in this article, or claim that may be made by its manufacturer, is not guaranteed or endorsed by the publisher.

## Abbreviations

TNF-α: tumor necrosis factor-alpha
DMEM: Dulbecco's Modified Eagle Medium
EVs: Extracellular vehicles
GI: gastrointestinal
IBS: Irritable Bowel Syndrome
IBD: Inflammatory Bowel Disease
BSA: Bovine Serum Albumin
SEM: Scanning Electron Microscope
TEM: Transmission Electron Microscopy
PMA: Phorbol Myristate Acetate
DLS: Dynamic Light Scattering.
